# Low prevalence of seasonal influenza viruses in Hong Kong, 2022

**DOI:** 10.1111/irv.13123

**Published:** 2023-03-17

**Authors:** Gannon C. K. Mak, Stephen S. Y. Lau, Kitty K. Y. Wong, Angela W. L. Lau, Derek L. L. Hung

**Affiliations:** ^1^ Microbiology Division, Public Health Laboratory Services Branch Centre for Health Protection, Department of Health Hong Kong Special Administrative Region People's Republic of China

**Keywords:** Hong Kong, Influenza A Virus, Prevalence

The seasonal influenza activity has been dramatically reduced worldwide due to COVID‐19 control measures.^1^, ^2^ Only sporadic influenza cases have been detected in Hong Kong from 2020 to 2022. In the present study, we report the investigation results of all the strains of influenza isolated in 2022.

Viral culture and characterization were routinely performed as described previously.^3^ As the number of influenza cases was low, we performed genetic characterization for all virus isolates, as per the established protocol. Clades designation was based on the European Centre for Disease Prevention and Control (ECDC) report.^4^

Between January and December 2022, a total of 60 influenza viruses were isolated and characterized (Table 1). Of the positive specimens, 71.7% (43/60) were collected between November and December. Overall, influenza A H3 (80%, 48/60) was most commonly detected, followed by influenza H1 (11.7%, 7/60) and influenza B (8.3%, 5/30). The full length of the HA gene for these 60 cases was sequenced and was submitted to GISAID database. Only a single clade was observed for influenza A H1 and influenza B, respectively. B/Yamagata lineage has not been detected in Hong Kong since 2020. For influenza A H3, two clades were detected. Of the 48 strains, seven of them belonged to clade 3C.2a1b.2a.1, which were all detected in June and July. From August 2022 onward, all cases belonged to clade 3C.2a1b.2a.2. Antigenically, all except three isolates characterized by haemagglutination inhibition (HAI) assay using the 2021–2022 WHO influenza reagent kit were having titre within fourfold of the corresponding H3 antigen control strain, A/Tasmania/503/2020. Those three isolates were influenza A H3 belonging to clade 3C.2a1b.2a.2 (Appendix A). The HAI assay results for them showed 8‐ to 16‐fold lower titre than the H3 antigen control. Sequencing of these isolates showed two unique mutations in the HA1 region, T135K and S145N, that were not found in the others. This substitution pattern has been reported by ECDC recently as well.^4^ The T135K substitution is of particular interest; it results in the loss of a glycosylation motif at a receptor‐binding antigenic epitope, potentially facilitating immunological escape. Previously reported circulating in 2016–2019, T135K mutation was associated with reduced neutralization titer^5^ and lower vaccine effectiveness.^6^ Recently, a significant proportion of genetically characterized H3 influenza virus in British Columbia in the early 2022–23 influenza season carried T135K mutation, and all were from clade 2a.2.^7^ In our study, T135K viruses were only detected sporadically between October and November, and its prevalence in the influenza season would be further observed.

**TABLE 1:** The number of influenza cases detected and characterized in the present study.

Month	Total	Influenza cases			
		H1	H3		B
		6B.1A.5a.2	3C.2a1b.2a.1	3C.2a1b.2a.2	V1a.3a.2
Jan	1	0	0	0	1
Feb	0	0	0	0	0
Mar	0	0	0	0	0
Apr	0	0	0	0	0
May	1	0	0	1	0
Jun	7	0	6	0	1
Jul	1	0	1	0	0
Aug	4	2	0	2	0
Sep	0	0	0	0	0
Oct	3	0	0	3	0
Nov	19	3	0	13	3
Dec	24	2	0	22	0
Total	60	7	7	41	5

Influenza activity in Hong Kong remained to be low in 2022. Various COVID‐19 control measures such as the universal masking policy were still in force at the time of writing this report.^8^ It is expected that the influenza immunity among the public was reduced due to low virus circulation in the past 3 years.^9^ A recent study predicting the burden of influenza in the upcoming seasons estimated there would be a 10%–60% increase in the population susceptibility to influenza, which might lead to a maximum of one‐ to fivefold rise in peak magnitude and one‐ to fourfold rise in epidemic size for the upcoming 2022–23 influenza season.^10^ The recommended H3 vaccine strain in 2022–2023 has been updated to 3C.2a1b.2a.2. Though the recent data from British Columbia reported vaccine effectiveness were below 50% among clade 2a.2 with T135K, the difference from those in the same clade without T135K was not statistically significant,^7^ and its actual impact remains to be studied. Ongoing surveillance for influenza as well as other respiratory viruses would be important to enable us realize their circulation pattern in the post‐SARS‐CoV‐2 pandemic time.

## AUTHOR CONTRIBUTIONS

**Gannon C.K. Mak:** Conceptualization; data curation; formal analysis; investigation; methodology; writing—original draft; writing—review and editing. **Stephen S.Y. Lau:** Investigation. **Kitty K.Y. Wong:** Investigation. **Angela W.L. Lau:** Investigation. **Derek L.L. Hung:** Writing—original draft; writing—review and editing.

## CONFLICT OF INTEREST STATEMENT

None.

### PEER REVIEW

The peer review history for this article is available at https://www.webofscience.com/api/gateway/wos/peer-review/10.1111/irv.13123.

## Data Availability

None.

## APPENDIX A

The three H3 isolates with hemagglutination inhibition (HAI) assay titre more than fourfold lower than the antigen control.

**Table:**

Isolate	Our lab number	Sex	Age	Date collected	Specimen type	HAI results			GISAID accession number
						Test strain	Antigen control strain	Fold change	
A/HK/16/2022	VB22019244	M	42 y	24/10/2022	Throat and nasal swab	80	1280	16	EPI_ISL_15809023
A/HK/17/2022	VB22019245	M	3 y	24/10/2022	Nasal swab	160	1280	8	EPI_ISL_15809024
A/HK/33/2022	VC22009967	M	15 y	14/11/2022	NPS + Throat swab	160	2560	16	EPI_ISL_16853057

NPS, nasopharyngeal swab.
